# Do Infants Attribute Moral Traits? Fourteen-Month-Olds' Expectations of Fairness Are Affected by Agents' Antisocial Actions

**DOI:** 10.3389/fpsyg.2018.01649

**Published:** 2018-09-07

**Authors:** Luca Surian, Mika Ueno, Shoji Itakura, Marek Meristo

**Affiliations:** ^1^Department of Psychology and Cognitive Sciences, University of Trento, Trento, Italy; ^2^Department of Psychology, Kyoto University, Kyoto, Japan; ^3^Department of Psychology, University of Gothenburg, Gothenburg, Sweden

**Keywords:** infants, fairness, distributive justice, moral development, moral judgment

## Abstract

We investigated whether and how infants link the domains of harm, help and fairness. Fourteen-month-old infants were familiarized with a character that either helped or hindered another agent's attempts to reach the top of a hill. Then, in the test phase they saw the helper or the hinderer carrying out an equal or an unequal distribution toward two identical recipients. Infants who saw the helper performing an unequal distribution looked longer than those who saw the helper performing an equal distribution, whereas infants who saw the hinderer performing an unequal distribution looked equally long than those who saw the hinderer performing an equal distribution. These results suggest that infants linked the hindering actions to a diminished propensity for distributive fairness. This provides support for theories that posit an early emerging ability to attribute moral traits to agents and to generate socio-moral evaluations of their actions.

**RESEARCH HIGHLIGHTS**

- Infants expect agents that previously helped another agent to perform egalitarian distributions, but they do not generate such expectation about agents that previously hindered another agent.

- This ability to link hindering and distributive actions is important because it may help the development of reasoning about agents' stable moral traits.

- Results provide support for recent theories on early social evaluation skills and they constraint theories on the acquisition of moral competence.

The developmental origins of socio-moral evaluations may be seen well before the kindergarten age. At 15–24 months, infants prefer agents that distribute resources equally, rather than unequally (Geraci and Surian, 2011; Burns and Sommerville, 2014; Surian and Franchin, 2017a) and by 9–10 months they expect resources to be distributed equally (Meristo et al., 2016; Ziv and Sommerville, 2017). Infants look longer when they see an agent distributing the available resources unequally rather than equally to similar recipients (Sloane et al., 2012; Sommerville et al., 2013), and by 24 months their expectations are guided by agents' merit and group membership (Sloane et al., 2012; Surian and Franchin, 2017b; Bian et al., 2018) and are associated to their altruistic sharing of a preferred toy (Schmidt and Sommerville, 2011; Ziv and Sommerville, 2017; Sommerville, 2018). Infacts' reactions to distributive events are not due to perceptual factors or expectations about agents' affiliative behavior (Meristo et al., 2016). They expect fair and unfair agents to be differently praised and admonished (DesChamps et al., 2016) or rewarded and punished (Meristo and Surian, 2013, 2014).

The early emergence of moral cognition is also revealed by studies on how infants react at agents that help or hinder others. Infants were presented animated scenarios or stage shows with agents that helped or hindered others' attempts to reach the top of a hill (e.g., Kuhlmeier et al., 2003; Hamlin et al., 2007, 2013), or live puppet shows involving a character that helped or prevented another agent from opening a box, or that returned a ball to someone who dropped it, rather than running away with it (e.g., Hamlin and Wynn, 2011; Hamlin, 2013). Infants' preference for helpers has been found in many studies, using a variety of stimuli and procedures (for a meta-analysis, Margoni and Surian, 2018). They also spontaneously help others, suggesting an understanding and concern for others' goals, intentions and needs (e.g., Dunfield and Kuhlmeier, 2010).

While there is now a growing body of evidence revealing infants' ability to evaluate hindering, helping and distributive actions, we do not know yet if they are also able to represent any link between these domains of actions. In this study we presented infants with agents that first helped or hindered another agent and then saw the helper or the hinderer performing either a fair or an unfair distribution. If infants take agents' helping or hindering behavior as a cue to their propensity to be fair or unfair, they should react differently to the distributive actions performed by the helper and the hinderer.

## Methods

### Participants

Thirty-two healthy full-term infants from Japanese-speaking families participated (age range: 14 months 19 days −15 months 25 days; *M* = 15 months 4 days; 16 female, 16 male). An additional 8 infants were tested but excluded from the sample because they were inattentive during the familiarization phase (*n* = 4), inattentive during the test phase (*n* = 3), or because of technical error (*n* = 1). Sample size was determined by similarity with most previous relevant studies. The infants recruited for the experiment were registered with Kyoto University Infant Research Fellow Program. We contacted their parents, explained them the outline of the experiments and their main purpose and obtained their written consent. The study was conducted according to Code of Ethics and Conduct of The Japanese Psychological Association.

### Materials and procedure

During the test session infants were seated on the parent's lap in a dimly lit and quiet booth 50–70 cm away from a 17-in.-monitor used to display the stimuli. The caretakers were asked to turn their head away from the screen and not to communicate with the infants during the testing. Infants' looking behavior was recorded and analyzed using a Tobii T60 (Tobii Technology, Sweden) corneal reflection near infrared Eye Tracker. Each testing session began with a 5-points infant calibration procedure.

**Figure 1:**
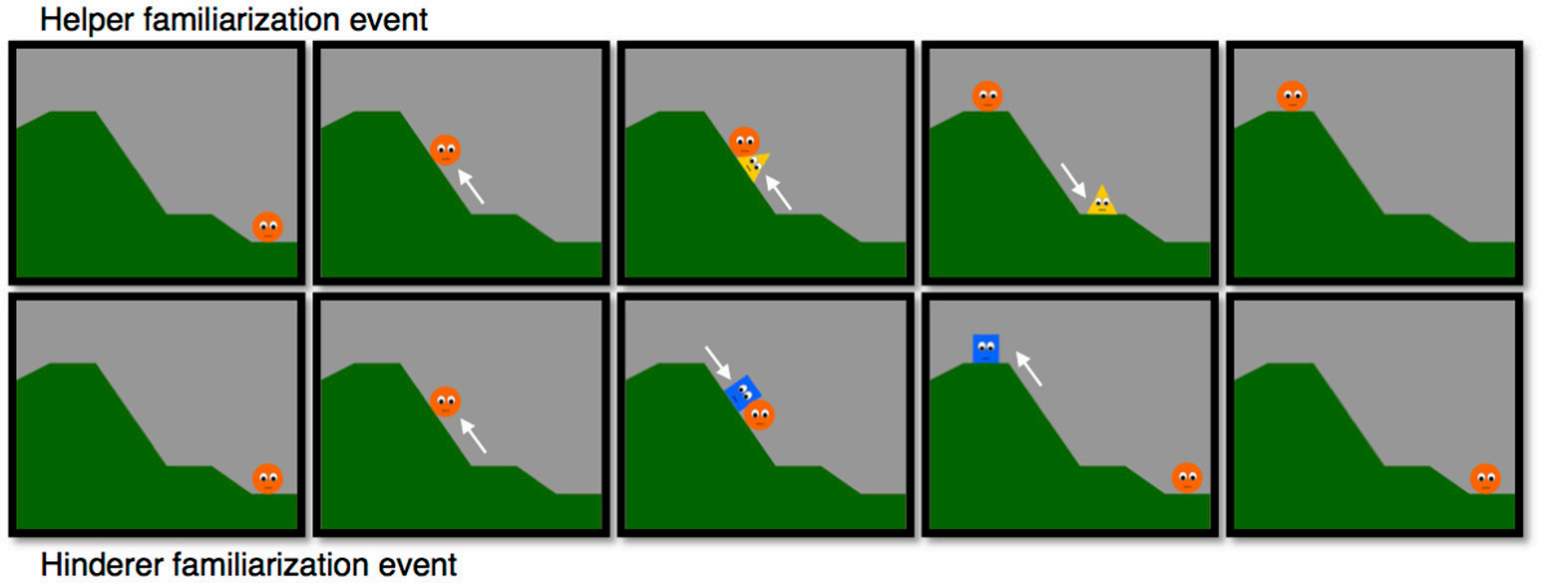
Selected frames from the familiarization events in the helper and hinderer condition.

Sixteen infants were assigned to one of two conditions, the helper and hinderer conditions. In both conditions infants saw four familiarization events followed by one test event. In the familiarization phase all infants saw two “helper events” and two “hinderer events.” In the *helper* familiarization events, infants first saw an agent, the “climber,” entering the screen from the right side at the bottom of a hill (see Figure 1). The climber then climbed to the lower plateau, and rotated itself slightly for 2 s, then attempted twice to reach the upper plateau, each time falling back to the lower plateau. Then the helper entered the display from the lower right, moved up the incline and bumped the climber twice, each time pushing it farther up until the climber reached the upper plateau. The climber then remained stationary at the top of the hill, while the helper moved back to the bottom of the hill and left the screen. In the “hinderer familiarization event,” the hinderer entered from the upper left, moved downward and bumped the climber twice, each time pushing it farther down. The climber then remained stationary, while the hinderer moved back on top of the hill and then exited.

**Figure 2:**
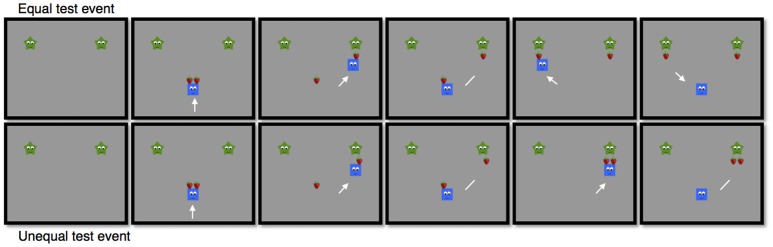
Selected frames from the test events with equal or unequal distribution.

The familiarization phase was followed by the *test phase*. In the *helper* condition infants saw the helper distributing two strawberries to two identical green stars-shaped characters (see Figure 2). The test event started with the two stars present, one on the left side and one on the right side in the upper part of the screen. Then, the helper entered from the right or the left side carrying two red strawberries and gave them to the stars. Half the infants saw an equal distribution and the other half saw an unequal distribution. At the end the helper stayed in the middle of the screen.

The *hinderer* condition started with the same familiarization phase used in the helper condition, but in the test phase the distributor of the strawberries was the hinderer instead.

We fully counterbalanced across the participants: (1) identity of the helper/hinderer (circle vs. triangle), (2) order of familiarization events (Help-Hinder-Hinder-Help vs. Hinder-Help-Help-Hinder), (3) side of the delivery of the first strawberry in the test event (Left vs. Right), and (4) test event (Equal vs. Unequal distribution), resulting in 16 different sessions. Infants had to follow at least three familiarization events to be included in the final analyses. The dependent measure was the time the infant spent looking at the still picture at the end of the test movie, until he or she looked away for at least 2.5 consecutive s, after having looked for at least 2.5 s.

## Results

Preliminary analyses assessed the effects of order of familiarization events (Help, Hinder, Hinder, Help vs. Hinder, Help, Help, Hinder) and identity of the helper and the hinderer (Square vs. Triangle), and found they had no main effect on looking times at the test trials, nor there was a significant interaction between such factors and the type of test event (equal vs. unequal distribution).

Looking times in the final test event were analyzed in a 2 × 2 ANOVA with condition (helper or hinderer) and test event (equal or unequal) as between-subject factors. The analyses showed a main effect for test event, *F*_(1, 31)_ = 7.08, *p* = 0.013, η^2^ = 0.20, and significant condition x test event interaction effect, *F*_(1, 31)_ = 10.00, *p* = 0.003, η^2^ = 0.28.

Planned contrast revealed a significant difference, with longer looks at unequal test events (*M* = 26.60 s, *SD* = 9.94) compared to the equal test events (*M* = 9.35 s, *SD* = 4.92), *t*_(14)_ = 4.40, *p* = 0.001, η^2^ = 0.58, in the helper condition, but not in the hinderer condition (Equal: *M* = 17.92 s, *SD* = 10.76; Unequal: *M* = 16.04 s, *SD* = 5.27), *t*_(14)_ = 0.45, *p* = 0.663, η^2^ = 0.01).

## Discussion

Infants were first presented with agents that carried out either a helping or hindering action and then they saw the same agents performing a fair or an unfair distribution. We found that infants looked longer at the unfair compared to the fair distribution performed by the helper, but looked equally long at the equal and unequal distributions performed by the hinderer.

This suggests that in the helper condition infants generated and maintained the default expectations about agents' fairness that have been shown in several previous studies (e.g., Schmidt and Sommerville, 2011; Sloane et al., 2012; Meristo et al., 2016; Ziv and Sommerville, 2017), but they canceled such expectations in the hinderer condition. The fact they looked equally long at the two types of distributions performed by the hinderer suggest that they did not generate an expectation opposite to the default expectation. At present, we do not know why this is the case. We suggest that infants may refrain from generating negative expectations about the agents' future actions and this bias could be the root of a phenomenon recently discovered in the adult literature, namely the bias to represent agents as possessing morally virtuous selves (De Freitas et al., 2017). This gives raise also to an alternative explanation for the present results: suppose that infants expected, by default, that agents would act helpfully toward other agents. When they saw the helper, infants left their default expectations unchanged. By contrast, when they saw the hinderer, they may have tagged that agent as one that behaves inconsistently. This alternative account differs from the one we proposed at the beginning because it is not committed to inconsistency just in morally valenced behavior, but in behavior more generally.

The present results support the claim that infants may be able to attribute a goodness trait linking the domains of fairness and prosocial actions. An ERP study that employed the same stimuli used here suggests that this tendency is preserved in adults (Ishikawa et al., 2017).

How deep and stable is this representation? One possibility, the “early concept view,” is that infants have already developed a rudimentary concept of *good agent* that includes features about agents' helping attitudes as well as their propensity to behave fairly (Uhlmann et al., 2015). An alternative possibility, the “simple mismatch view,” claims that the present results were simply driven by the mismatch in the values attributed to the actions performed by the helping agent in the familiarization and test phases, with no role played by prior expectations about how an helping agent will or will not behave in a distributive context. The present findings are consistent with both of these interpretations. Note, however, that the simple mismatch view would predict significant results also in the hinderer condition. By contrast, the early concept view does not make such prediction since the features used to diagnose agents' goodness and badness are different. Also, in both accounts the underpinned processes require an attribution of opposite values to helping and unfair actions, consistently with current proposals on infants' socio-moral competence (Baillargeon et al., 2015).

Further studies are needed to see whether the present results generalize to other instances of helping/hindering actions and infants' inferences can run from observing distributive behavior to expecting helping or hindering actions. It would also be interesting to test whether the same results can be found if infants, in the familiarization phase, do not see both a helper and hinderer, but just one of these two agents. This would be helpful in deciding whether they need to see both types of characters in order to evaluate them and generate behavioral expectations. Other crucial goals for future studies would be to investigate the duration of memories about agents' pro- and anti-social tendencies.

The ability to rely on information about agents' hindering or helpful actions to generate expectations about their distributive fairness has, potentially, far-reaching implications. Most importantly, it suggests that infants display an early ability to attend and evaluate actions in order to construct a stable socio-moral representation of agents. This ability may provide the initial basis for the acquisition of an explicit conception of moral goodness.

## Ethics statement

The study was conducted according to Code of Ethics and Conduct of The Japanese Psychological Association. The research project was approved by the Ethical Committee of the University of Trento. The parents of the infants who participated in the experiment gave their written consent.

## Author contributions

LS, MM, and SI designed the study; MM prepared the experimental materials; MM and MU carried out the data collection and the statistical analyses; LS and MM wrote the first draft of the manuscript; SI and MU provided revisions.

### Conflict of interest statement

The authors declare that the research was conducted in the absence of any commercial or financial relationships that could be construed as a potential conflict of interest.
